# Establishment of a Rapid and Effective *Agrobacterium*-Mediated Genetic Transformation System of *Oxalis triangularis* ‘Purpurea’

**DOI:** 10.3390/plants12244130

**Published:** 2023-12-11

**Authors:** Yun Xiao, Wanli Tuo, Xuexuan Wang, Baomin Feng, Xinyu Xu, Sagheer Ahmad, Junwen Zhai, Donghui Peng, Shasha Wu

**Affiliations:** 1 College of Landscape Architecture and Art, Fujian Agriculture and Forestry University, Fuzhou 350002, China; 13347398023@163.com (Y.X.); twl13395088391@163.com (W.T.); candicewxx@163.com (X.W.); 18120872516@163.com (X.X.); sagheerhortii@gmail.com (S.A.); zhai-jw@163.com (J.Z.); fjpdh@126.com (D.P.); 2 Plant Immunity Center, Fujian Agriculture and Forestry University, Fuzhou 350002, China; baomin2006@126.com

**Keywords:** *Oxalis triangularis* ‘Purpurea’, *Agrobacterium* mediated, transient expression, genetic transformation

## Abstract

*Oxalis triangularis* ‘Purpurea’ has significant ornamental value in landscaping. There is a critical necessity to elucidate the gene functions of *O. triangularis* ‘Purpurea’ and dissect the molecular mechanisms governing key ornamental traits. However, a reliable genetic transformation method remains elusive. In this study, our investigation revealed that various transformation parameters, including recipient material (petioles), pre-culture time (2–5 days), acetosyringone (AS) concentration (100–400 μM), *Agrobacterium* concentrations (OD_600_ = 0.4–1.0), infection time (5–20 min), and co-culture time (2–5 days), significantly impacted the stable genetic transformation in *O. triangular* ‘Purpurea’. Notably, the highest genetic transformation rate was achieved from the leaf discs pre-cultured for 3 days, treated with 200 μM AS infected with *Agrobacterium* for 11 min at OD_600_ of 0.6, and subsequently co-cultured for 3 days. This treatment resulted in a genetic transformation efficiency of 9.88%, and it only took 79 days to produce transgenic plants. Our transformation protocol offers advantages of speed, efficiency, and simplicity, which will greatly facilitate genetic transformation for *O. triangular* ‘Purpurea’ and gene function studies.

## 1. Introduction

*Oxalis triangularis* ‘Purpurea’, commonly called false shamrock, is a color-leafed ornamental flowering plant belonging to the *Oxalis* family (Oxalidaceae). It is renowned for the distinctive characteristics of leaflets, including unique shape, rich color, and intriguing nyctinastic movement. This plant is widely appreciated and is extensively used in flower beds, gardens, and as potting plants for indoor adornment [1]. The leaves of *O. triangularis* ‘Purpurea’ are palmately compound, composed of three leaflets that resemble inverted triangles with entire margins. They are clustered at the base of the plant. The purplish-red hue of the leaf gives the name *O. triangular* ‘Purpurea’ [1,2]. One of its most notable features is that the three leaflets exhibit a fascinating response to light changes. These leaflets unfold in high-light conditions during the daytime and close in low-light environments at night [3]. Several genes associated with leaflet coloration have been identified in *O. triangularis* ‘Purpurea’ leaves. Luo et al. [3] proposed that MYB113 and TTG2 may participate in anthocyanin biosynthesis. However, a reliable transformation procedure for *O. triangularis* ‘Purpurea’ is currently a bottleneck to functionally characterize the genes dictating the aforementioned important and intriguing traits.

*Agrobacterium*-mediated genetic transformation is a widely used technique for plant transformation. Compared to other methods, such as gene gun bombardment, *Agrobacterium*-mediated transformation offers several advantages, including ease of operation, cost-effectiveness, low copy of gene integration, and the capacity to transfer large DNA fragments [4]. Horsch et al. [5] developed a genetic transformation protocol starting from leaf discs in tobacco, petunia, and tomato. The improved leaf disc method includes explant materials such as cotyledonary leaves, rachis, leaf sections, stem segments, petioles, immature embryos, and others [6,7,8]. It has been widely adapted for various ornamental plants to generate transgenic lines [9,10], such as *Lilium* L. [11], *Petunia hybrida* [12], and *Rosa hybrida* [13]. Genetic transformation usually relies on the construction of a regeneration system, which often takes a long time and turns out to be difficult. The process of genetic transformation generally involves multiple stages, with the transfer of T-DNA into the plant cell being a critical step [14]. Therefore, assessing the transient expression of reporter genes in different experimental variants can help optimize the efficiency of this stage. Transient expression is a fast and effective method for studying the function of plant genes [15]. High transient expression by *Agrobacterium* indicates that the selected explants are more receptive and potentially suitable for the stable transgene. Additionally, the plant expression vector pCAMBIA1304 contains the hygromycin phosphotransferase gene (*HPT*), which is a selection marker that facilitates the quick selection of transgenic tissues or plants [16]. It also includes β-Glucuronidase (*GUS*), which is useful for establishing transformation systems for plants. *GUS* is a reporter gene that offers high sensitivity [17]. It can catalyze X-Gluc into a blue-colored product, which is widely used for reporting the success of genetic transformation [18].

In this study, we established an effective *Agrobacterium*-mediated transient expression and genetic transformation system of *O. triangularis* ‘Purpurea’ using its petiole as a transformation recipient. We optimized the parameter combination for efficient transient expression and established transgenic plants from the petiole of *O. triangularis* ‘Purpurea’. Via *GUS* staining and multiple rounds of PCR screening of transgenic plants, it was confirmed that the *GUS* gene had been integrated into the plant. Therefore, this work serves as a crucial step for the functional characterization of genes related to the ornamental traits of *O. triangularis* ‘Purpurea’. Furthermore, our findings have the potential to offer valuable insights for genetic engineering and breeding research not only in *O. triangularis* ‘Purpurea’ but also in other species of the *Oxalis* genus.

## 2. Results

### 2.1. Different Recipient Explants of O. triangularis ‘Purpurea’ Showed Different Transformation Efficiency

**Figure 1:**
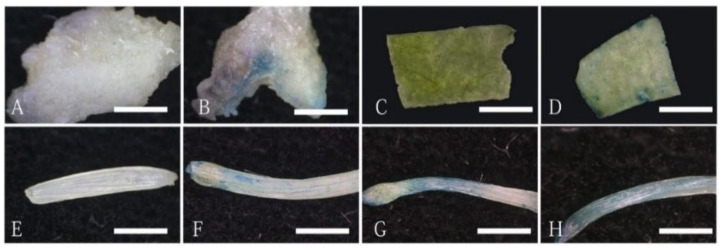
The *GUS* staining results of different recipient explants. (**A**,**B**) The *GUS* staining of the callus tissue without (**A**) or with (**B**) the *Agrobacterium*-mediated *GUS* transient expression; (**C**,**D**) the *GUS* staining of leaf tissues without (**C**) or with (**D**) the *Agrobacterium*-mediated *GUS* transient expression. (**E**–**H**) The *GUS* staining of the petiole without (**E**) or with (**F**–**H**) the *Agrobacterium*-mediated *GUS* transient expression. Scale bars = 5 mm.

For *Agrobacterium*-mediated plant transformation, different tissues or organs could serve as explants. However, their efficiency to be transformed differs significantly. In this study, we tested different explants, including leaf, petiole, and calluses from *O. triangularis* ‘*Purpurea*’ for their transformation efficiency. We used the *GUS* reporter to check the transient expression efficiency and found that this could be a good indicator of transformation success. After co-culturing the explants with *Agrobacterium* carrying pCAMBIA1304 for three days, the *GUS* staining was performed, and the transient expression rate of *GUS* was calculated. Among the explants tested, the petioles showed the best transient expression rate of *GUS* (Table 1), and the *GUS* transient expression rate of petioles was much higher than that in the callus and leaves. The blue staining area of the callus was relatively small (Figure 1B) and concentrated in one region, with a transient expression rate of 15.62%. A few blue spots appeared in the injured areas and near the veins of leaves (Figure 1D), and the transient expression rate of *GUS* was 12.5%. The blue staining can be observed at both ends and inside the petiole (Figure 1F–H), with a transient *GUS* expression rate of 56.81%. Therefore, this study used petioles as recipients to optimize key factors for transient expression and stable transformation.

### 2.2. The Effects of Antibiotic Concentrations on Petiole Growth

**Figure 2:**
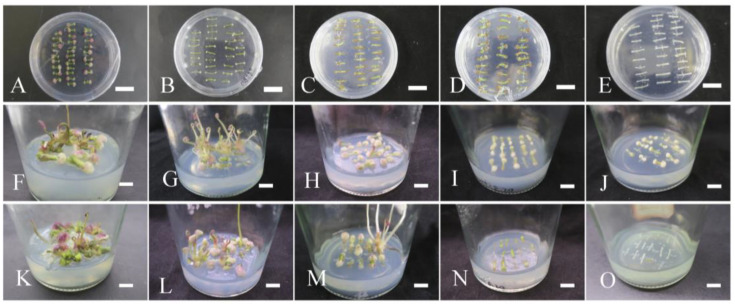
Effects of different concentrations of antibiotics on the growth of petiole for 30 days. (**A**–**E**) The petiole growth status in different concentrations of hygromycin (**A**, 0.0 mg·L^−1^; **B**, 0.4 mg·L^−1^; **C**, 0.6 mg·L^−1^; **D**, 0.8 mg·L^−1^; **E**, 1.0 mg·L^−1^). (**F**–**J**) Petiole growth status in different concentrations of carbenicillin (**F**, 0 mg·L^−1^; **G**, 100 mg·L^−1^; **H**, 200 mg·L^−1^; **I**, 300 mg·L^−1^; **J**, 400 mg·L^−1^). (**K**–**O**) Petiole growth status in different concentrations of cefotaxime (**K**, 0 mg·L^−1^; **L**, 100 mg·L^−1^; **M**, 200 mg·L^−1^; **N**, 300 mg·L^−1^; **O**, 400 mg·L^−1^). Scale bar = 1 cm.

The petioles were cultured on an M1 medium containing hygromycin at different concentrations, and the results are shown in Table S1. The callus induction rate decreased sharply with the increase in hygromycin concentration. After 30 days of culture, the callus induction rate of petiole on 0 mg·L^−1^ hygromycin was the highest, reaching 100%, followed by that of 0.4 mg·L^−1^ hygromycin, with callus induction rate inducing to 92.22%. The browning rate was only 7.78% (Figure 2A). When the petiole was cultured on hygromycin at 0.6 and 0.8 mg·L^−1^, a small amount of callus was generated at both ends of the petiole in the early stage, but later, the callus induction rate dropped to 61.11% and 34.44%. The petiole browning rate rose to 38.88% and 65.55%, respectively (Figure 2B,C). However, when the petioles were cultured in the medium with hygromycin above 1 mg·L^−1^, the petiole failed to induce callus, and bleaching or browning began on the 20th day and was completed on the 30th day (Figure 2D,E). It was concluded that 1 mg·L^−1^ of hygromycin was the lethal concentration for petiole. Therefore, a semi-lethal concentration of hygromycin (0.8 mg·L^−1^) was selected as the concentration for primary screening for 30 days and then transferred to a culture medium containing 1 mg·L^−1^ hygromycin for 30 days for secondary screening.

We found that the growth of petioles without carbenicillin (Carb) and cefotaxime (Cef) was not inhibited, callus was induced on the 10th day, and petiole differentiation began on the 20th day. When the concentration of Cef was approximately between 100 and 200 mg·L^−1^, the induction of petiole callus was not affected, but when the concentration reached 300–400 mg·L^−1^, the growth of petioles was inhibited, and browning and whitening occurred. However, Carb of 100–200 mg·L^−1^ had no significant effect on the petiole growth, while Carb with a concentration of 300–400 mg·L^−1^ inhibited the induction rate of petiole callus but did not cause damage to the petiole (Figure 2). To reduce the inhibitory effect on petiole growth, we chose 200 mg·L^−1^ Carb as the optimal concentration for the selection culture since 100–400 mg·L^−1^ Carb can inhibit the growth of *Agrobacterium*.

### 2.3. Optimizing Key Parameters by Combining Transient Expression Rate and Resistant Callus Rate

**Figure 3:**
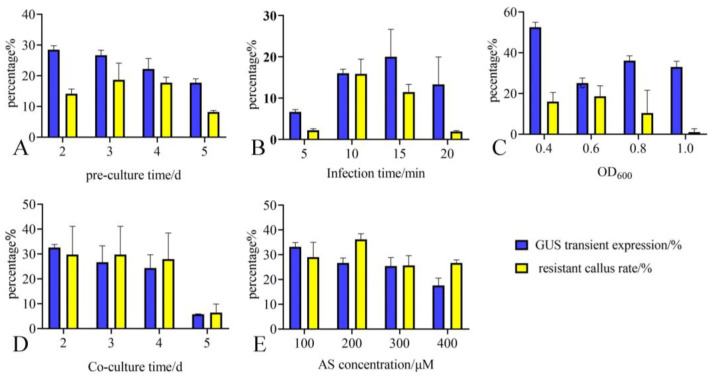
Parameters that affect genetic transformation efficiency. (**A**) The effect of pre-culture time on transformation efficiency. (**B**) The effect of infection time on transformation efficiency. (**C**) The effect of bacterial concentration OD_600_ on transformation efficiency. (**D**) The effect of co-culture time on transformation efficiency. (**E**) The effect of AS concentration on transformation efficiency. All data are represented as the mean ± standard deviation of the triplicate samples. d: day.

The data analysis here referred to the analysis of “Key Transformation Factors” by Yan et al. [19]. Pre-culture of plants is beneficial for the invasion of *Agrobacterium* and the recovery of plants from wounding. Based on Figure 3A and Figure S1, the transient expression level gradually decreased with the extension of pre-culture time. The transient expression rate and resistant callus rate obtained after 2–4 days of pre-culture are the highest. When the pre-culture time reaches 5 days, both ends of the petiole have started to expand, and the surrounding cells have shifted from dedifferentiation to differentiation, which is not conducive to the integration of exogenous DNA. As pre-culture time increased, the rate of transient expression decreased. Therefore, it is recommended that the pre-culture time for petiole genetic transformation of *O. triangularis* ‘Purpurea’ should not exceed 5 days.

The *GUS* transient expression rate exhibited an increasing trend with the extension of time when the infection time was 5–20 min (Figure 3B and Figure S1). When the infection time was 5 min, the *GUS* transient expression rate was the lowest (only 6.67%), while it reached its highest point if the infection time was extended to 10–15 min. The resistant callus rate increased first and then decreased. When the concentration of *Agrobacterium* suspension was OD_600_ = 0.6 and infection lasted for 10 min or 15 min, the resistant callus rate was the highest (15.85% and 11.46%, respectively). When the infection time was 5 min or 20 min, the resistant callus rate was low, 1.00% or 1.91%, respectively. However, infection for 20 min seriously damaged the petioles, and some petioles showed bleaching and browning. Therefore, this study recommends 10–15 min as an appropriate time of infection for petiole genetic transformation.

The concentration of bacterial suspension is related to the growth state and violence of *Agrobacterium*, both affecting the transformation efficiency. When the concentration of *Agrobacterium* was OD_600_ = 0.4, the transient expression rate of *GUS* was the highest (52.54%). When the concentration of *Agrobacterium* was 0.6, 0.8, and 1.0, the transient expression rate of *GUS* was lower (33%, 36.11%, and 33.01%, respectively). When OD_600_ was 0.4–0.6, the resistant callus rates were high, accounting for 16.16% and 18.70%, respectively. When the OD_600_ value reached 1.0, the resistant callus rate was low, only 1.07% (Figure 3C and Figure S1). The higher the concentration of *Agrobacterium*, the more difficult the washing process was. So, we recommend OD_600_ = 0.4–0.8 as the optimal concentration for genetic transformation.

After 1–2 days of culture, the surface of some petioles did not change significantly. They were still green, and the volume was not expanded. After infection by *Agrobacterium*, some petioles turned brown. Most of the petioles were killed by *Agrobacterium* in the subsequent washing and screening process. When the co-culture time was 2–4 days, the *GUS* transient expression rate and resistant callus rate were high, which were 33.33%, 26.67%, and 24.39% and 25.44%, 22.49%, and 16.67%, respectively (Figure 3D and Figure S1). After 5 days of co-culture, the long-term co-culture led to *Agrobacterium* over-grow, making the petiole completely submerged by *Agrobacterium* biofilm. Even if *Agrobacterium* was inhibited during the screening and culture process, the growth of the petiole was also seriously affected by *Agrobacterium* and could not recover. Therefore, the co-culture time should not exceed 5 days.

The results showed that the transient expression of *GUS* decreased with the increase in AS concentration (Figure S1). When the concentration of AS was 100, 200, and 300 μM, the transient expression rate of *GUS* was high, accounting for 33.24%, 26.67%, and 25.49%, respectively. When the concentration of AS was 400 μM, the transient expression rate of *GUS* was the lowest, only 17.54%. In another observation, the resistance callus rate increased first and then decreased. When the concentration of AS was 200 μM, the resistant callus rate was high (36.19%). When the concentration of AS was 300 and 400 μM, the resistant callus rate was low, 25.56% and 26.67%, respectively (Figure 3E). So, it is recommended that the 100–200 μM is an appropriate AS concentration.

### 2.4. Regeneration of Hygromycin-Resistant Plants

**Figure 4:**
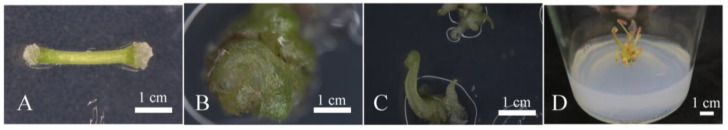
*Agrobacterium*-mediated transformation of petioles in different stages of *Oxalis triangularis* ‘Purpurea’. (**A**) The resistant petiole began to swell; (**B**) Induced resistant callus from resistant petioles; (**C**) The resistant shoots formed by differentiation; (**D**) Regenerated seedlings of the hygromycin-resistant plants.

In this study, the genetic transformation of *O. triangularis* ‘Purpurea’ was completed via the use of the petiole. After 1 week of screening and cultivation on M3 media, both ends of the resistant petiole began to swell (as shown in Figure 4A). After 4 weeks of screening, the resistant petiole induced callus and began to differentiate (Figure 4B). The untransformed petiole gradually turned brown or died, and it was subsequently removed. The hygromycin-resistant petioles were then transferred to the M4 medium. After 2 weeks, the callus differentiated into buds (Figure 4C). Finally, after 4 weeks, residual seeds were observed (Figure 4D).

### 2.5. Determination of Genetic Transformation Efficiency

#### 2.5.1. *GUS* Staining of the Hygromycin-Resistant Plants of *O. triangularis* ‘Purpurea’

**Figure 5:**
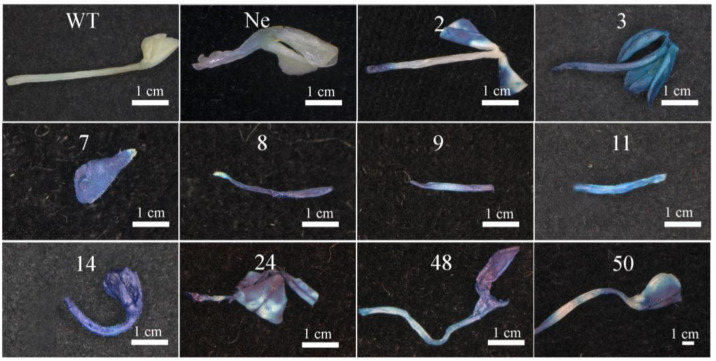
Identification of transgenic lines by genotyping and *GUS* staining. Blue coloring was observed in the transformed plants, whereas the WT and untransformed plants only showed minimal dot coloring or even white. WT: Wild-type plants, Ne: Negative plants (untransformed plants), numbers 2, 3, 7, 8, 9, 11, and 14: Positive plants from treatment 2; number 24: Positive plants from treatment 18; numbers 48 and 50: Positive plants from treatment 7.

After screening in a hygromycin-containing medium, 55 hygromycin-resistant plants were selected from all 20 treatments. Two leaves or petioles were randomly selected from the fully unfolded leaves of each resistant plant. One leaf or petiole was used for *GUS* staining. As shown in Figure 5, compared with the wild type (WT and untransformed plants), the *GUS* staining result of transgenic plants was dark blue across the entire petiole and leaves, while the *GUS* staining result of the control group was white.

#### 2.5.2. PCR Identification of the Hygromycin-Resistant Plants of *O. triangularis* ‘Purpurea’

**Figure 6:**
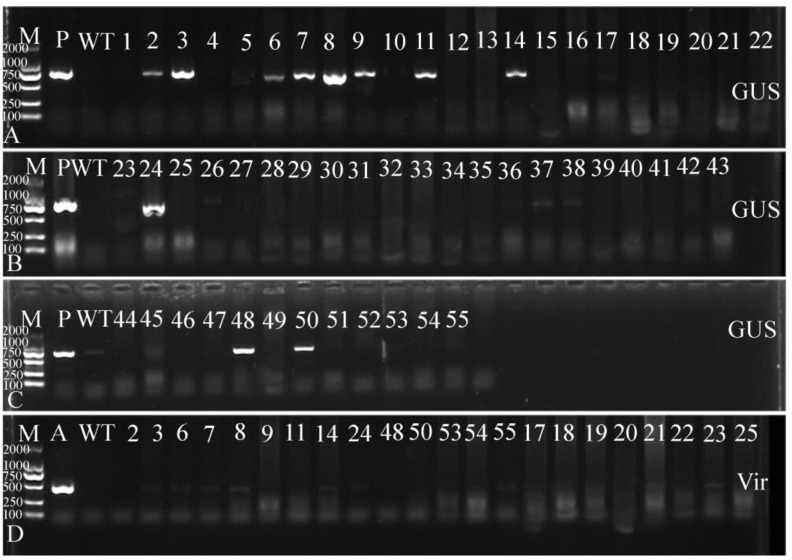
Identification of transgenic lines by genotyping PCR. (**A**–**C**) PCR identification of the *GUS* (723 bp) in hygromycin-resistant lines. M: DNA marker 2 Kb, P: Plasmid pCAMBIA1304; WT: Wild-type plant; numbers 1–55: hygromycin-resistant plants. (**D**) The *Vir* gene amplification (450 bp). (All positive transgenic plants and some non-transgenic plants were displayed, and others had no bands amplified). A: the *Agrobacterium;* WT: Wild-type; numbers 2, 3, 6, 7, 8, 9, 11, and 14: Positive plants from treatment 2; number 24: Positive plants from treatment 18; numbers 48 and 50: Positive plants from treatment 7; other numbers are untransformed plants.

Another leaf of each hygromycin-resistant plant was used to extract genomic DNA and for PCR identification by specific primers (*GUS*-F/R and *Vir*-F/R). The PCR products with the expected molecular weight were also sent for Sanger sequencing. Further PCR identification was conducted on hygromycin-resistant plants, and 11 plants showed expected PCR products of *GUS* (723 bp confirmed by Sanger sequencing), indicating that the *GUS* gene had been integrated into the plant genome (Figure 6). In addition, to rule out *Agrobacterium* contamination, PCR of *Vir* gene (present in *Agrobacterium* but not in the T-DNA fragment) was performed and no amplified fragments of the *Vir* gene were observed in all transgenic plants, although a 450 bp fragment could be amplified from the positive control (the *Agrobacterium*), clearly indicating that the obtained transgenic plants were not false positive (the complete PCR and RT-PCR gel electrophoresis images are in Figures S2–S4). Finally, 11 transgenic plants were identified, with the highest genetic transformation efficiency obtained from treatment 2 (Table 2). Seventeen resistant plants and eight positive plants were obtained, with a transformation efficiency of 9.88% and a positive rate of 47.06%.

#### 2.5.3. Detection of Transgene Expression by RT-PCR

**Figure 7:**
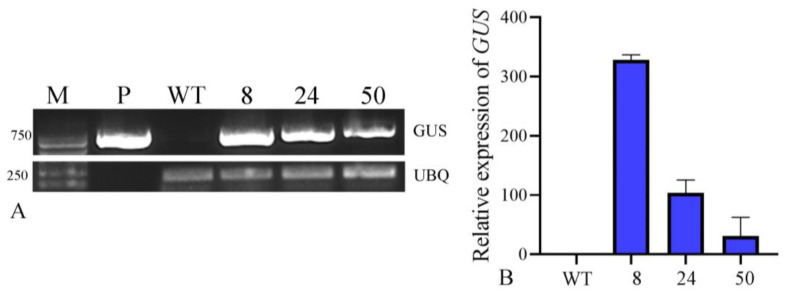
Detection of transgene expression by RT-PCR and RT-qPCR. (**A**) RT-PCR analysis of *GUS* expression in wild-type plants and transgenic lines. M: Marker 2000, P: pCAMBIA1304, WT: Wild-type; numbers 8, 24, and 50: transgenic lines from treatment 2, treatment 18, and treatment 7, respectively; (**B**) RT-qPCR analysis of *GUS* transcriptional levels in WT plants and transgenic lines. WT: wild-type; numbers 8, 24, and 50: transgenic plants from treatment 2, treatment 18, and treatment 7, respectively.

To further confirm the transgenic events, we performed semi-quantitative RT-PCR and qPCR to examine the transgene’s mRNA abundance and found that the RNA levels detected were consistent with the *GUS* staining results (Figure 7A). The expected amplification product of *GUS* (723 bp) was observed in all three selected transgenic plants and the positive control, pCAMBIA1304, but not in the wild type. Furthermore, the results indicated that transgenic plants exhibited substantially high levels of *GUS* expression, and the relative expression level of *GUS* is consistent with the *GUS* staining intensity. (Figure 7B).

## 3. Discussion

In recent years, the *Agrobacterium*-mediated genetic transformation has been applied to the functional characterization of genes in ornamental plants and developed as an important research tool [20]. Xu et al. [21] reported the genetic transformation of the exogenous gene chalcone synthase (*CHS*) in the leaves of *O. triangularis* ‘Purpurea’. Initially, we attempted to employ the leaves as recipients for genetic transformation, but we were unsuccessful because the leaves were sensitive to hygromycin and had a low ability for regeneration.

Successful genetic transformation requires a good recipient with potent regeneration abilities and tolerance to *Agrobacterium* [22]. Therefore, we investigated which might be effective recipient material for the genetic transformation of *O. triangularis* ‘Purpurea’ and tested the leaves, petioles, and callus as recipients. The findings showed that the efficiency of transient expression in the leaves and callus was low and that bacterial removal after co-cultivation was challenging because of the callus’ three-dimensional shape and its substantial contact area with *Agrobacterium*. The surface of the callus exhibited variable degrees of browning and mortality after becoming infected with *Agrobacterium*, leading to a lengthier regeneration period. The petiole is composed of the normal xylem, phloem, and intermediate cambium. The cambium cells offer advantages, including resistance to *Agrobacterium* and excellent cell division and plant regeneration capabilities [23,24], and have advantages, such as resistance to *Agrobacterium*. Sheng et al. [25] believed that this may be related to the support of vascular tissue for growth and development. After comparing different types of explants as the recipients of the transgene, we found that the petiole of *O. triangularis* ‘Purpurea’ is the best.

The transgenic events in this study were consistently confirmed by three independent methods: genotyping PCR of genomic DNA, *GUS* staining, and qPCR analysis of transgene expression. Our study also suggested that the *GUS* reporter is a good indicator of successful transgene events and could be used to guide the optimization of transgene procedures.

Effective selection of transformed cells and tissues is a crucial step in plant genetic transformation [26,27]. We empirically found that hygromycin is a better selective antibiotic for the genetic transformation of the petioles of *O. triangularis* ‘Purpurea’. Antibiotic resistance genes such as neomycin phosphotransferase II (*NPT II*) and hygromycin B (*HPT*) have been widely used for transgenic plant selection. The sensitivity of different plant materials to these antibiotics differs significantly. However, this is a key factor for screening and needs to be determined in the first place [28]. It was found that the petioles of *O. triangularis* ‘Purpurea’ are more sensitive to hygromycin. The appropriate concentration of hygromycin screening not only ensured sufficient induction efficiency but also facilitated the screening of resistant regenerated seedlings. When screening with hygromycin, the callus and adventitious buds induced by the failed explants exhibited various symptoms: (1) petiole growth inhibition; (2) petiole browning, albinism, and even death; and (3) petiole inability to induce callus and inability to differentiate normally. Based on these obvious phenomena, it was easy to identify regenerated plants that have failed in transformation. The results indicated that a major drawback of kanamycin compared to hygromycin is that many species exhibit high levels of natural tolerance to this antibiotic, which may lead to many untransformed false-positive individual plants [29]. Therefore, we used hygromycin as a screening agent in our current work.

After co-cultivation, it is vital to remove excess *Agrobacterium*. Thus, appropriate antibacterial agents must be used to suppress *Agrobacterium* growth while not interfering with plant growth. The types and concentrations of antibacterial agents have different effects on different recipients, so it is necessary to explore the minimum concentration at which antibacterial agents have the smallest impact on the growth of receptors. The results ultimately indicated that Carb had less effect on receptor growth than Cef, making it more suitable as an antibacterial agent.

According to previous reports, the *Agrobacterium* strains and concentrations [30,31], pre-culture time [32], co-culture time [33], the concentration of AS [34], infection time [35,36], and medium type have significant effects on genetic transformation efficiency [37]. The pre-culture might make the explant cells more susceptible to *Agrobacterium*. It can also reduce the damage of *Agrobacterium* to the explant, thereby improving the transformation efficiency. The infection time correlates with the degree of damage caused by *Agrobacterium* to the recipient tissues, and the bacterial concentration might affect the total virulence of *Agrobacterium*. Co-culture is key for transient expression and genetic transformation, during which the T-DNA transfer and the foreign gene expression begin [38]. AS can activate the *Vir* region genes of *Agrobacterium*, promoting the transfer and integration of T-DNA into plant cells [39]. These factors collectively affect the transformation efficiency. The preferred conditions for the transformation of lily callus include bacterial OD_600_ = 0.4–0.8, the infection time ranging from approximately 15 to 30 min, and AS 100 μM [8], co-cultured for 3 days [40,41]. Most of the lotus seeds were inoculated with *Agrobacterium* (OD_600_ = 0.6) for 1–5 min and cultured for 2–7 days [42]. This study showed that pre-culture time was 2–4 d, the OD_600_ was 0.4–0.8, and the infection time was between 10 and 15 min, the addition of 100–200 μM AS during the *Agrobacterium* activation stage and co-culture time was 2–4 d and gave better genetic transformation efficiency of *O. triangularis* ‘Purpurea’.

Among these factors, the concentration of *Agrobacterium* and infection time are closely related to expression efficiency [43]. The infection involved the attachment of *Agrobacterium* to the recipient cells. However, if the concentration of the infection solution is too high or the infection time is too long, *Agrobacterium* will excessively adhere, leading to *Agrobacterium* contamination and browning or necrosis of the recipient tissues, reduced transformation efficiency, and even killing the plant material [44,45]. On the contrary, if the concentration of the infection solution is too low or the infection time is too short, the *Agrobacterium* adhesion is insufficient, reducing transformation efficiency. It can be seen that the infection time is too long or too short, resulting in lower transient expression. In this study, excess *Agrobacterium tumefaciens* caused significant damage to the explants. When the infection time reached 20 min, although *GUS* transient expression was 13.8%, the resistant callus rate sharply decreased after screening with 0.8 mg·L^−1^ hygromycin, only 1.91%. As the infection time prolonged, the petiole would undergo bleaching, leading to a decrease in the survival rate of the recipient material and thus reducing the transient expression. When the infection time was 5 min, the transient expression and genetic transformation rate were both low, which is consistent with that for *Codonopsis pilosula* [46].

This work also proved that adding AS during the *Agrobacterium* activation stage and co-culturing can significantly improve transformation efficiency. The genetic transformation procedure established in this study has the advantage of high transformation efficiency, ranging from 1.02% to 9.88% (Table 2). The transformation efficiency is comparable to that of other ornamental flower plants, such as *Chrysanthemum morifolium* Ramat (the transformation efficiency is between 1% and 8%) [47,48,49], *Broccoli oleracea* var. italica (2.7–6.4%) [27], *B. oleraceae* var Botrytis (7–13.6%) [50], and *L. pumilum* DC Fisch (5.7–13.0%) [51].

Finally, the use of a simple culture medium reduced the workload and increased efficiency. Throughout the tissue culture period, only one callus induction medium was required, which can induce both callus and differentiation. The formula for this medium was simple. By streamlining the experimental process and simplifying the medium formula, this approach not only reduced costs and workload but also enhanced practical operation repeatability, providing a higher potential for genetic transformations.

## 4. Materials and Methods

### 4.1. Plant Materials and Medium

Healthy petioles of *O. triangularis* ‘Purpurea’ were uniformly chosen from the greenhouse. They were thoroughly washed under running water for three hours, followed by rinsing with 75% alcohol for 45 s on a clean workbench. After that, the petioles were cleaned with sterile water for one minute and disinfected by shaking them in an explant-disinfectant (the effective ingredient is 2–3% NaClO) from Beijing Huayue Biological (Beijing, China) for eight minutes. Finally, they were rinsed twice with sterile water for one minute each time. The petioles were cut into 5 ± 0.5 mm and placed onto the M1 medium to induce callus for 30 days. After that, a part of the callus from the petioles was transferred to a proliferation medium, which included Murashige-Skoog (MS, Solarbio, Beijing, China) supplemented with 1 mg·L^−1^ N6-benzyladenine (6-BA, Solarbio, Beijing, China), 0.5 mg·L^−1^ a-naphthaleneacetic (NAA, Solarbio, Beijing, China), 30 mg·L^−1^ glucose(Solarbio, Beijing, China), 7 g·L^−1^ agar(Solarbio, Beijing, China), and 0.05 g·L^−1^ activated carbon(Solarbio, Beijing, China). When the callus became loose and granular, it was used for transient expression. Another part of the callus from the petioles continued to differentiate for 60–120 days for genetic transformation.

Pre-culture medium (M1): MS supplemented with 0.5 mg·L^−1^ NAA and 1 mg·L^−1^ 6-BA. The co-culture medium (M2): MS supplemented with 0.5 mg·L^−1^ NAA, 1 mg·L^−1^ 6-BA, and 200 μM AS. The primary screening medium (M3): MS supplemented with 0.5 mg·L^−1^ NAA, 1 mg·L^−1^ 6-BA, 200 mg·L^−1^ Carb (Solarbio, Beijing, China), and 0.8 mg·L^−1^ hygromycin (Yeasen, Shanghai, China). The screening medium (M4): MS supplemented with 0.5 mg·L^−1^ NAA, 1 mg·L^−1^ 6-BA, 100 mg·L^−1^ Carb, and 1.0 mg·L^−1^ hygromycin. All the media contained 30 g·L^−1^ sucrose, 0.05 g·L^−1^ activated carbon, and 7 g·L^−1^ Agar. The pH of the medium was adjusted to 5.7–5.8 before sterilization, and it was sterilized at 121 °C and 104 kPa for 20 min. The infection solution consisted of 4.74 g·L^−1^ MS, 30 g·L^−1^ sucrose. The pH was adjusted to 5.8. The solution was autoclaved, and 100–400 μM AS (Macklin, Shanghai, China) was added after cooling it down. The LB medium included 10 g·L^−1^ tryptone, 10 g·L^−1^ NaCl (Solarbio, Beijing, China), 5 g·L^−1^ yeast powder (Solarbio, Beijing, China), and 15 g·L^−1^ Agar (Solarbio, Beijing, China). The pH was adjusted to 7.0 before autoclaving. The plant culture conditions were: 12 h·d^−1^ light cycle, light intensity of 2500–3000 Lx, 25 ± 2 °C temperature, and 50–70% humidity.

### 4.2. Plant Expression Vector and Agrobacterium Strain

The pCAMBIA1304 vector contains the 35S promoter, the β-glucuronidase (*GUS*) gene as a reporter gene, and the hygromycin phosphotransferase (*HPT*) gene as a selective marker gene. The vector was donated by Professor Songjun Zeng, South China Botanical Garden, Chinese Academy of Sciences. *Agrobacterium* GV3101 was purchased from Sangon Biotech (Shanghai, China).

The glycerol stock of *Agrobacterium* stored at −80 °C was taken out and streaked on an LB plate. After 2–3 days of culture at 28 °C, a single bacterial colony and placed on 1 mL of LB medium containing 50 mg·L^−1^ Kanamycin (Solarbio, Beijing, China) and 25 mg·L^−1^ Rifampicin (Yeasen, Shanghai, China). Incubated at 28 °C and 200 rpm for 12 h, 2 μL were taken for PCR identification, and after PCR confirmation, the culture was used to inoculate 50 mL of LB liquid medium (with 50 mg·L^−1^ Kanamycin and 25 mg·L^−1^ Rifampicin) and shaken at 200 rpm and 28 °C until the culture turned orange (logarithmic growth period), centrifuged at room temperature at 4000 rpm for 10 min, and the bacteria were collected and resuspended in the transformation solution. The OD_600_ of the bacterial suspension was adjusted to 0.4–1.0 with the infection solution, which was kept in the dark for 2–3 h before transformation.

### 4.3. Determination of Transformation Receptor

After more than two subcultures, the leaves, petioles, and callus of robust sterile seedlings were used as transient expression transformation receptors. After three days of pre-cultivation, they were infected with *Agrobacterium tumefaciens* solution with OD_600_ = 0.6 for 11 min. After three days of co-cultivation, they were washed with sterile water and then stained overnight with *GUS* to calculate the *GUS* staining rate.

### 4.4. Determination of Selection Pressure

To screen transgenic plants and inhibit the over-growth of *Agrobacterium* during selection, sensitivity tests for hygromycin (Hyg), carbenicillin (Carb), and cefotaxime (Cef) were conducted on the wild-type petioles of *O. triangularis* ‘Purpurea’. In the trial experiment, hygromycin concentrations ranging from 10 mg·L^−1^ to 50 mg·L^−1^ were used, but the final result showed that all petioles bleached and died. Therefore, after adjustment, the new petiole was cut into 5 ± 0.5 mm and transferred to an M1 medium (30 petioles per medium) containing low hygromycin concentrations (0, 0.4, 0.6, 0.8, and 1.0 mg·L^−1^). Similarly, Cef (0, 100, 200, 300, and 400 mg·L^−1^) and Carb (0, 100, 200, 300, and 400 mg·L^−1^) were added to the M1 medium (15 petioles per medium). Each of the three independent experiments had 30 petioles, repeated three times. After 30 days, the browning rate, bleaching rate, and callus induction rate of petioles were counted, and the growth under different concentrations of hygromycin and antibiotics was recorded.

### 4.5. Optimization of the Parameters for Genetic Transformation

Optimization of pre-culture time (treatment 1–4): After 2, 3, 4, or 5 days of culturing petioles in the dark on the M1 medium, the petioles were inoculated with a bacterial suspension (OD_600_ = 0.6), incubated for 11 min, and the concentration of AS was adjusted to 200 μM. After incubation, the residual bacteria on the surface of the petioles were drained and co-cultured on the M2 medium for 3 days. After that, 45 petioles were selected for *GUS* staining to measure *GUS* intensity grade and calculate the transient expression rate. The remaining 90 petioles were transferred to the M3 medium, and the resistant callus rate was calculated after 30 days. After that, the resistant calli were transferred to the M4 medium for cultivation until resistant plants were differentiated.

Optimization of infection time (treatment 5–8): After the petioles were cultured on the M1 medium and in darkness for three days, the concentration of bacterial suspension was adjusted to OD_600_ = 0.8, and different infection times (5, 10, 15, or 20 min) were tested. The concentration of AS was 200 μM, and the co-culture time was 3 days.

Bacterial concentration optimization (treatment 9–12): This optimization was carried out in a similar way as above. All parameters were the same except for the following: After three days of culture in darkness on the M1 medium, the petioles were incubated with different bacteria suspensions (OD_600_ = 0.4, 0.6, 0.8, or 1.0) for 11 min. The concentration of AS was 200 μM, and the co-culture time was 3 days.

Optimization of co-culture time (treatment 13–16): After three days of culture in darkness on the M1 medium, the petioles were incubated with bacteria suspension (OD_600_ = 0.4) for 11 min. The concentration of AS was 200 μM. Different co-culture times (2, 3, 4, or 5 days) were tested for the effects on transformation.

Optimization of the AS concentration (treatment 17–20): After three days of culture in darkness on the M1 medium, the petioles were incubated with bacteria suspension (OD_600_ = 0.4) for 11 min. The co-culture time was set as 3 days. Different AS concentrations (100, 200, 300, or 400 μM) were tested for the influence on transformation efficiency.

### 4.6. The GUS Staining

The petioles were kept in a 1.5 mL centrifuge tube, and the 1 × *GUS* staining solution was added according to the kit instructions. The wild-type petioles were used as negative controls. The petioles were wrapped in foil and kept in the dark at 37 °C overnight and then rinsed with 75% alcohol until the wild-type petioles turned white. The staining was observed under a microscope, and the transient expression rate was calculated.

### 4.7. PCR Identification of Positive Plants

PCR identification: The total genomic DNA of the resistant plants was isolated by DNA purification kit (Tiangen, Beijing, China) for PCR identification, and the *GUS*-specific primers were designed according to the *GUS* gene sequence (*GUS* F: 5′-GTGAATCCGCACCTCTGG-3′; *GUS* R: 5′-ATCGCCGCTTTGGACATA-3′). *Agrobacterium*–specific primers were designed based on the sequence of the *Agrobacterium Vir* gene (*Vir* F: 5′-ATAGCCAGCACCTCTTGCAG-3′; *Vir* R: 5′-GCGGACAAAGTTATTGCGCT-3′). The amplification products were separated and analyzed by 1% agarose gel electrophoresis. PCR products of all positive plants were sent for Sanger sequencing (Sangon, Shanghai, China).

RT-qPCR identification: Three positive plants were randomly selected for gene expression analysis. Total RNAs were extracted from the leaves and petioles of transgenic plants and control non-transgenic plants following the protocol of plant RNA extraction kit (Vazyme, Nanjing, China.), and then gDNA was removed using FastPure gDNA-Filter Columns III. cDNA Synthesis was carried out using Hifair V one-step RT gDNA digest Super MIX (Yeasen, Shanghai, China.) from 1 μg RNA. The first strand cDNA was amplified into a specific 723 bp *GUS* fragment for genomic DNA amplification using the primer pairs (*GUS*-F: 5′-GCTCTACACCACGCCGAACAC-3’, *GUS*-R: 5’-GCCACCACCTGCCAGTCAAC-3’), while the primer pairs (*UBQ*-F: 5’-GCAGTTCTACAAGGTCGACG-3’, *UBQ*-R: 5’-GCCACACTTGCCACAGTAAT-3’) were used for the reference gene ubiquitin (*UBQ*) (450 bp) amplification. The RT-PCR reaction conditions were 98 °C for 3 min, 30 cycles of 98 °C for 10 s, 60 °C for 20 s, and 72 °C for 30 s, and then 72 °C for 5 min, saved at 4 °C. The volume of the RT-qPCR reaction system was 10 µL, including 5 µL Blue SYBR Master Mix (Yeasen, Shanghai, China), 0.2 µL 10 µM forward primers, 0.2 µL 10 µM reverse primer, 1 µL cDNA, and 3.6 µL ddH_2_O. The RT-qPCR was carried out in an ABI 7500 fast system, and the reaction procedure included 95 °C for 30 s, 40 cycles of 95 °C for 3 s, 60 °C for 20 s, and 72 °C for 30 s, other settings default to the instrument. The relative transcriptional expression levels of each gene were calculated by 2^−∆∆Ct^.

### 4.8. Data Analysis

Statistical analysis was carried out in Excel 2016 and SPSS 20.0. Duncan’s new complex range method was used to analyze the significant differences among samples. All data are presented as the means ± standard error (SE) of at least three independent experiments.

Browning rate = the number of browning petioles/total petiole number × 100%. Bleaching rate = the number of bleaching petioles/total petiole number × 100%. Callus induction rate = the callus number/total petiole number × 100%. *GUS* transient expression rate = the number of petioles stained blue/total number of petioles stained × 100%. Resistant callus rate = the number of resistant calli induced/total petiole numbers on selection medium × 100%. Genetic transformation rate = the number of confirmed transgenic plants/total numbers of petioles × 100%. Positive rate = the number of confirmed transgenic plants/numbers of resistant plants × 100%.

## 5. Conclusions

**Figure 8:**
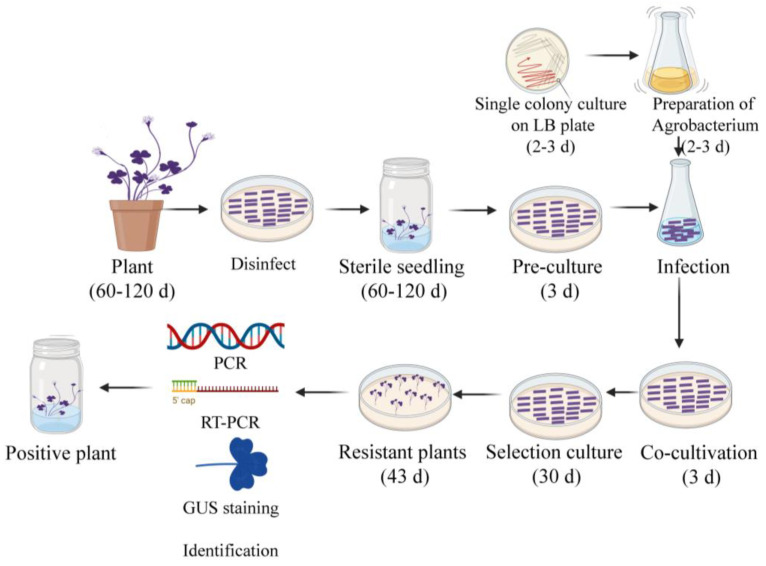
The flowchart of *Agrobacterium*-mediated genetic transformation of the petioles of *O. triangularis* ‘Purpurea’. The regeneration process includes disinfection, callus induction (10–20 days), differentiation (10 days), and maturation (30–90 days). The genetic transformation process includes pre-culture (3 days), co-culture (3 days), primary screening culture (30 days), and a second screening culture (43 days). It was drawn using BioRender. com. Note: Pre-culture and co-culture periods were placed in a dark environment.

*Agrobacterium*-mediated genetic transformation is a critical method for studying gene function. However, many plant species lack practical transformation procedures. This study established a reliable genetic transformation procedure by using the petiole of *O. triangularis* ‘Purpurea’ via an *Agrobacterium*-mediated method. It is the first time that such a system was established in this species. The transgenic events were confirmed by *GUS* staining, genotyping PCR coupled with Sanger sequencing, and qPCR. These methods collectively demonstrate the reliability of the procedure. This transformation method has high efficiency: the *GUS* reporter revealed that the transformation efficiency ranged from 1.02% to 9.88%. It takes as short as 79 days to obtain resistant plants (Figure 8).

In summary, this article reported an efficient, low-cost, and short-cycled transgene and plant regeneration method achieved by optimizing different transformation parameters, which will facilitate future research on the molecular and synthetic biology of *O. triangularis* ‘Purpurea’.

## Figures and Tables

**Table 1:** Different recipient explants show different *GUS* transient expression efficiency.

Recipient Explants	Total Number of Explants	*GUS* Positive Explants	*GUS* Transient Expression Rate
Petiole	44	25	56.82%
Leaf	40	5	12.5%
Callus	45	7	15.56%

**Table 2:** Identification of transgenic plants and transformation rate.

Treatment	Pre-Culture Time (d)	Co-Culture Time (d)	Bacterial Concentration (OD_600_)	Infection Time (min)	AS Concentration (μM)	Total Transformation Rate (%)
2	**3**	3	0.6	11	200	9.88
7	3	3	0.8	**15**	200	2.36
18	3	3	0.4	11	**200**	1.02

The bold, underlined font indicates the independent variables of each single-factor experiment.

## Data Availability

All relevant data can be found within the manuscript and its Supplementary Material.

## Supplementary Materials

The following supporting information can be downloaded at https://www.mdpi.com/article/10.3390/plants12244130/s1, Table S1: Effects of different hygromycin concentrations on petiole induction in callus; Figure S1: *GUS* transient expression results at different treatments. A-D: *GUS* staining results at different pre-culture times (2–5 days); E-H: *GUS* staining results of different OD_600_ (0.4–1.0); I-L: *GUS* staining results at different infection times (5–20 min); M-P: *GUS* staining results at different co-culture times (2–5 days); Q-T: A *GUS* staining results at different AS concentrations (100–400 μM); Figure S2: Identification of transgenic lines by genotyping PCR in hygromycin-resistant lines; Figure S3: RT-PCR analysis of *GUS* expression in Wild-type plants and transgenic lines; Figure S4: RT-PCR result of *UBQ* and *GUS* in Wild-type plants and transgenic lines.

## References

[1] Rosna M.T., Noraini M., Jamilah S.Y., Noorlidah A., Sadegh M. (2013). Synthetic seeds production and regeneration of *Oxalis triangularis* for mass propagation and conservation. J. Int. Environ. Appl. Sci..

[2] Rojas C., Díaz G., Montenegro O., Ferrada E. (2023). Occurrence of leaf rust disease in *Oxalis triangularis* caused by *Puccinia oxalidis* in Valdivia, Chile. Plant Dis..

[3] Luo B., Chen L., Chen G., Wang Y., Xie Q., Chen X., Hu Z. (2022). Transcription and metabolism pathways of anthocyanin in purple shamrock (*Oxalis triangularis* A. St.-Hil.). Metabolites.

[4] Xia P., Hu W., Liang T., Yang D., Liang Z. (2020). An attempt to establish an *Agrobacterium*-mediated transient expression system in medicinal plants. Protoplasma.

[5] Horsch R.B., Fry J.E., Hoffmann N.L., Wallroth M., Eichholtz D., Rogers S.G., Fraley R.T. (1985). A simple and general method for transferring genes into plants. Science.

[6] Baek S., Han J.E., Ho T.-T., Park S.Y. (2022). Development of hairy root cultures for biomass and triterpenoid production in *Centella asiatica*. Plants.

[7] Su W.B., Xu M.Y., Radani Y., Yang L.M. (2023). Technological development and application of plant genetic transformation. Int. J. Mol. Sci..

[8] Gupta V., Rahman L.-U. (2015). An efficient plant regeneration and *Agrobacterium*-mediated genetic transformation of *Tagetes erecta*. Protoplasma.

[9] Li M., Wang D., Long X., Hao Z., Lu Y., Zhou Y., Peng Y., Cheng T., Shi J., Chen J. (2022). *Agrobacterium*-mediated genetic transformation of embryogenic callus in a *Liriodendron hybrid* (*L.* Chinense × *L.* Tulipifera). Front. Plant Sci..

[10] Jin C.L., Dong L.Q., Wei C., Wani M.A., Yang C.M., Li S.C., Li F. (2023). Creating novel ornamentals via new strategies in the era of genome editing. Front. Plant Sci..

[11] Chen Y., Hou X., Zheng Y., Lyu Y. (2023). The establishment of a genetic transformation system and the acquisition of transgenic plants of oriental hybrid lily (*Lilium* L.). Int. J. Mol. Sci..

[12] Zvi M.M.B., Negre-Zakharov F., Masci T., Ovadis M., Shklarman E., Ben-Meir H., Tzfira T., Dudareva N., Vainstein A. (2008). Interlinking showy traits: Co-engineering of scent and colour biosynthesis in flowers. Plant Biotechnol. J..

[13] Zvi M.M.B., Shklarman E., Masci T., Kalev H., Debener T., Shafir S., Ovadis M., Vainstein A. (2012). PAP1 transcription factor enhances production of phenylpropanoid and terpenoid scent compounds in rose flowers. New Phytol..

[14] Chilton M.-D., Tepfer D.-A., Petit A., David C., Casse-Delbart F., Tempé J. (1982). *Agrobacterium rhizogenes* inserts T-DNA into the genomes of the host plant root cells. Nature.

[15] Canto T. (2016). Transient expression systems in plants: Potentialities and constraints. Adv. Exp. Med. Biol..

[16] Idnurm A., Bailey A.M., Cairns T.C., Elliott C.E., Foster G.D., Ianiri G., Jeon J. (2017). A silver bullet in a golden age of functional genomics: The impact of *Agrobacterium*-mediated transformation of fungi. Fungal Biol. Biotech..

[17] Mudunkothge J.S., Krizek B.A. (2014). The *GUS* reporter system in flower development studies. Flower Development. Methods in Molecular Biology.

[18] De Ruijter N.C.A., Verhees J., van Leeuwen W., van der Krol A.R. (2003). Evaluation and comparison of the *GUS*, *LUC* and *GFP* reporter system for gene expression studies in plants. Plant Biol..

[19] Yan R., Wang Z.P., Ren Y.M., Li H.Y., Liu N., Sun H.M. (2019). Establishment of efficient genetic transformation systems and application of CRISPR/Cas9 genome editing technology in *Lilium pumilum* DC. Fisch. and *Lilium longiflorum* White Heaven. Int. J. Mol. Sci..

[20] Qiu J., Sun S., Luo S., Zhang J., Xiao X., Zhang L., Wang F., Liu S. (2014). *Arabidopsis* AtPAP1 transcription factor induces anthocyanin production in transgenic *Taraxacum brevicorniculatum*. Plant Cell Rep..

[21] Xu T., Wu D., Yang X., Fu H., Wang J.G. (2010). Optimization of genetic transformation system of *Oxalis triangularis* ‘Purpurea’. Heilongjiang Sci..

[22] Tie W., Zhou F., Wang L., Xie W., Chen H., Li X., Lin Y. (2012). Reasons for lower transformation efficiency in indica rice using *Agrobacterium tumefaciens*-mediated transformation: Lessons from transformation assays and genome-wide expression profiling. Plant Mol. Biol..

[23] Alaguero-Cordovilla A., Sánchez-García A.B., Ibáñez S., Albacete A., Cano A., Acosta M., Pérez-Pérez J.M. (2021). An auxin-mediated regulatory framework for wound-induced adventitious root formation in tomato shoot explants. Plant Cell Environ..

[24] Liu S., Liu Y., Yang X., Tong C., Edwards D., Parkin I.A., Zhao M., Ma J., Yu J., Huang S. (2014). The *Brassica oleracea* genome reveals the asymmetrical evolution of polyploid genomes. Nat. Commun..

[25] Sheng X., Yu H., Wang J., Shen Y., Gu H. (2022). Establishment of a stable, effective and universal genetic transformation technique in the diverse species of *Brassica oleracea*. Front. Plant. Sci..

[26] Supartana P., Shimizu T., Shioiri H., Nogawa M., Nozue M., Kojima M. (2005). Development of simple and efficient in planta transformation method for rice (*Oryza sativa* L.) using *Agrobacterium tumefaciens*. J. Biosci. Bioeng..

[27] Chhikara S., Chaudhary D., Yadav M., Sainger M., Jaiwal P.-K. (2012). A non-tissue culture approach for developing transgenic *Brassica juncea* L. plants with *Agrobacterium tumefaciens*. In Vitro Cell. Dev. Biol. Plant.

[28] Zhang J., Wu J., Hao X., Xie Y., Lv K., Xu W. (2023). Establishment of a stable grape immature zygotic embryo-based genetic transformation system. Sci. Hortic..

[29] Wilmink A., Dons J. (1993). Selective agents and marker genes for use in transformation of monocotyledonous plants. Plant Mol. Biol. Rep..

[30] Wang X.H., Zhou F.L., Liu J.L., Liu W.Q., Zhang S.L., Li D.L., Song J.K., Wang R., Yang Y.J. (2021). Establishment of efficient callus genetic transformation system for *Pyrus armeniacaefolia*. Sci. Hortic..

[31] Chen J., Wang L., Chen J.B., Huang J., Liu F., Guo R., Yang L., Grabon A., Zhao K., Kong F.L. (2018). *Agrobacterium tumefaciens*-mediated transformation system for the important medicinal plant *Dendrobium catenatum* Lindl. In Vitro Cell. Dev. Biol. Plant.

[32] Yao J.L., Tomes S., Gleave A.P. (2013). Transformation of apple (*Malus× domestica*) using mutants of apple acetolactate synthase as a selectable marker and analysis of the T-DNA integration sites. Plant Cell Tissue Organ Cult..

[33] Xiao X., Li Y., Qin J.L., He Y., Cai W.Y., Chen Z.W., Xi L.Y., Zhang J.M. (2020). An optimized *Agrobacterium tumefaciens*-mediated transformation system for random insertional mutagenesis in *Fonsecaea monophora*. J. Microbiol. Methods..

[34] Singh P., Khan S., Kumar S., ur Rahman L. (2017). Establishment of an efficient *Agrobacterium*-mediated genetic transformation system in *Pelargonium graveolens*: An important aromatic plant. Plant Cell Tissue Organ Cult..

[35] Huang P., Xu M., Xia L., Qing Z., Tang Q., Liu W., Zeng J. (2017). Establishment of an efficient *Agrobacterium*-mediated genetic transformation method in *Macleaya cordata*. Sci. Hortic..

[36] Du Z.Z., Zong Q.Q., Gao H.F., Guo Q.Y., Liu T.G., Chen W.Q., Gao L. (2021). Development of an *Agrobacterium tumefaciens*-mediated transformation system for *Tilletia controversa* Kuhn. J. Microbiol. Methods.

[37] Hiei Y., Ishida Y., Komari T. (2014). Progress of cereal transformation technology mediated by *Agrobacterium tumefaciens*. Front. Plant Sci..

[38] Liu Z.Y., Ji J.J., Jiang F., Tian X.R., Li J.K., Gao J.P. (2022). Establishment of a genetic transformation system for *Codonopsis pilosula* callus. Plant Biotechnol..

[39] Chan M.T., Chang H.H., Ho S.L., Tong W.F., Yu S.M. (1993). *Agrobacterium*-mediated production of transgenic rice plants expressing a chimeric α-amylase promoter/β-glucuronidase gene. Plant Mol. Biol..

[40] Ogaki M., Furuichi Y., Kuroda K., Chin D., Ogawa Y., Mii M. (2008). Importance of co-cultivation medium pH for successful *Agrobacterium*-mediated transformation of *Lilium× formolongi*. Plant Cell Rep..

[41] Nakano M., Otani M. (2020). Plant regeneration and *Agrobacterium*-mediated genetic transformation systems in liliaceous ornamental plants. Plant Biotechnol..

[42] Liu Y., Lu X., Zhang H., Li S., Li Z. (2023). Establishment of a highly efficient in vitro propagation system of *Diospyros lotus*. Forests.

[43] Yasmin A., Debener T. (2010). Transient gene expression in rose petals via *Agrobacterium* infiltration. Plant Cell Tissue Organ Cult..

[44] Wang D., Zhu C.X., Zheng C.C., Wen F.J. (2002). Optimization of factors affecting genetic transformation of potato via *Agrobacterium tumefaciens*. J. Shandong Agric. Univ. (Nat. Sci.).

[45] Sutradhar M., Mandal N. (2023). Reasons and riddance of *Agrobacterium tumefaciens* overgrowth in plant transformation. Transgenic Res..

[46] Yang J., Yang X., Li B., Lu X., Kang J., Cao X. (2020). Establishment of in vitro culture system for *Codonopsis pilosula* transgenic hairy roots. 3 Biotech.

[47] Shinoyama H., Mitsuhara I., Ichikawa H., Kato K., Mochizuki A. (2015). Transgenic chrysanthemums (*Chrysanthemum morifolium* Ramat.) carrying both insect and disease resistance. Acta Hortic..

[48] Naing A.H., Ai T.N., Jeon S.M., Lim S.H., Kim C.K. (2016). An efficient protocol for *Agrobacterium*-mediated genetic transformation of recalcitrant chrysanthemum cultivar Shinma. Acta Physiol. Plant.

[49] Firsov A., Mitiouchkina T., Shaloiko L., Pushin A., Vainstein A., Dolgov S. (2020). *Agrobacterium*-mediated transformation of chrysanthemum with artemisinin biosynthesis pathway genes. Plants.

[50] Zhao Y., Yang D., Liu Y., Han F., Li Z. (2023). A highly efficient genetic transformation system for broccoli and subcellular localization. Front. Plant Sci..

[51] Song S., Yan R., Wang C., Wang J., Sun H. (2020). Improvement of a genetic transformation system and preliminary study on the function of *LpABCB21* and *LpPILS7* based on somatic embryogenesis in *Lilium pumilum* DC. Fisch. Int. J. Mol. Sci..

