# Supplementary material for: Establishment of a Rapid and Effective Agrobacterium-Mediated Genetic Transformation System of Oxalis triangularis ‘Purpurea’
Source: Plants (Basel). 2023 Dec 11;12(24):4130. doi: 10.3390/plants12244130 (PMC10747433; doi:10.3390/plants12244130)
Supplement: Supplementary file 1 [file plants-12-04130-s001.zip › plants-2696132-supplementary.pdf]

Supplementary File

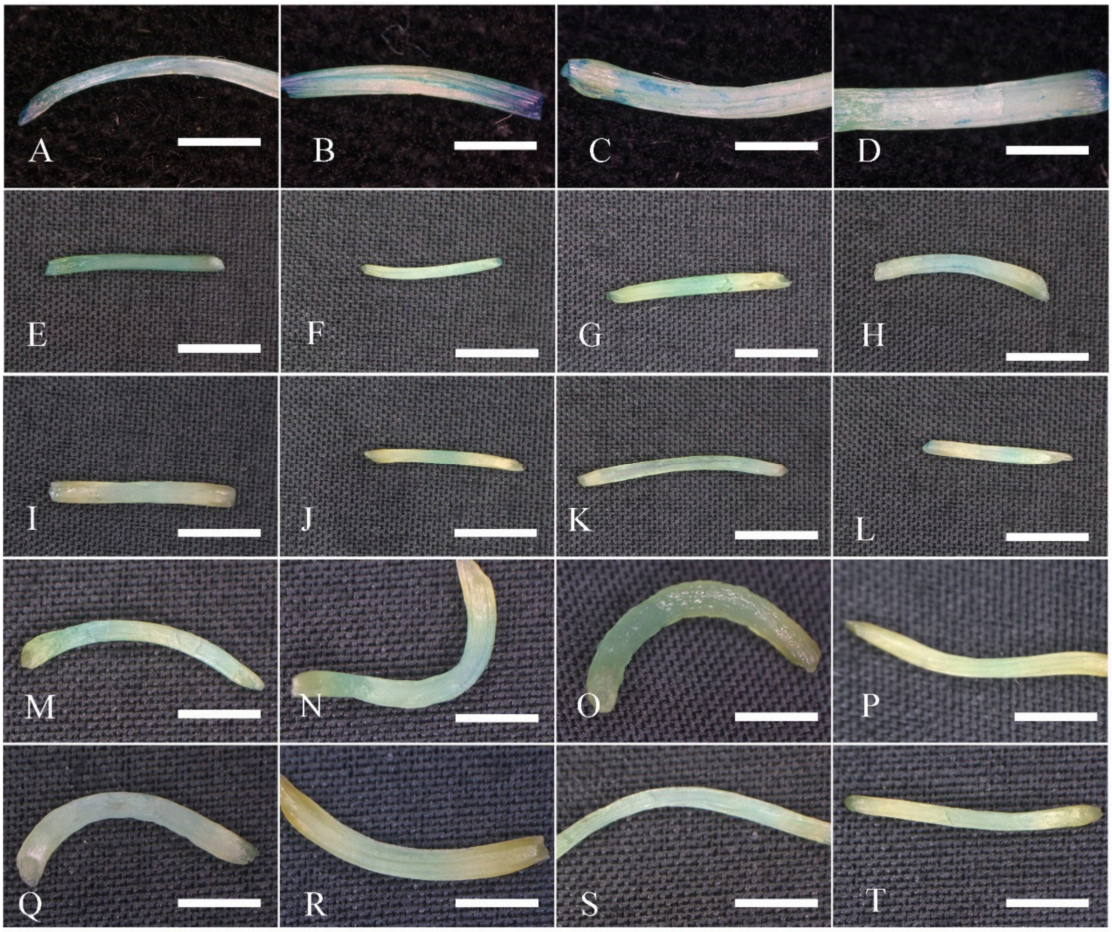

Figure S1 *GUS* transient expression results at different treatments

A-D: *GUS* staining results at different pre-culture times (2~5 days); E-H: *GUS* staining results of different OD_{600} (0.4~1.0); I-L: *GUS* staining results at different infection times (5~20 minutes); M-P: *GUS* staining results at different co-culture times (2~5 days); Q-T: A *GUS* staining results at different AS concentrations (100~400 μM). Scale bars = 5 mm.

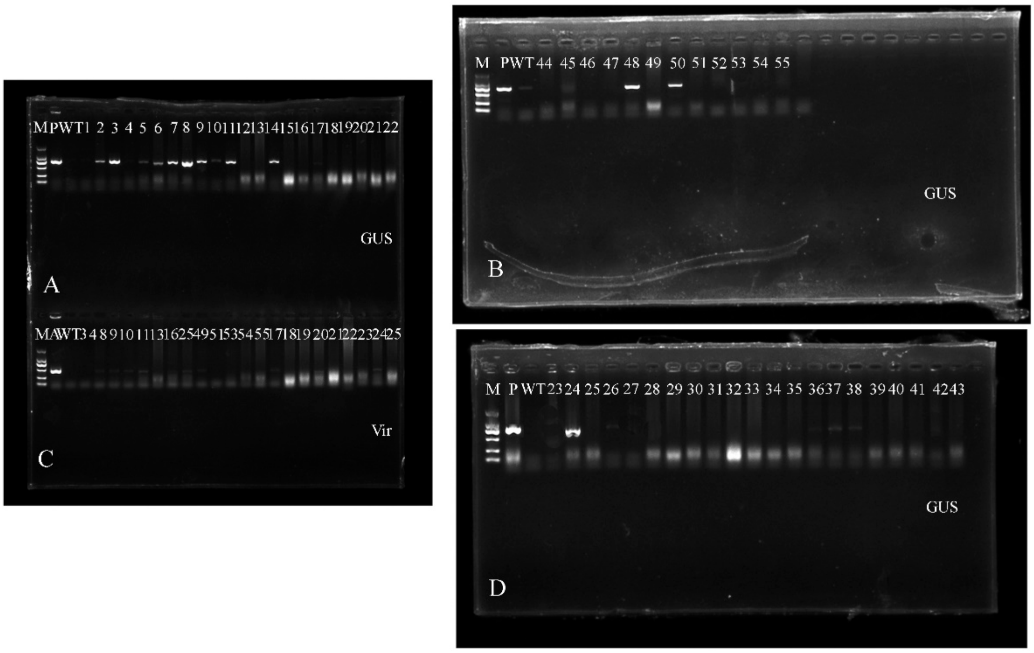

Figure S2 Identification of transgenic lines by genotyping PCR in hygromycin-resistant lines. (A-B, D) PCR identification of the *GUS* (723 bp) in hygromycin-resistant lines. M: DNA marker 2 Kb, P: Plasmid pCAMBIA1304; WT: Wild type plant; numbers 1-55: hygromycin-resistant plants. (C) The *Vir* gene amplification (450 bp). (all positive transgenic plants and some non-transgenic plants were displayed, and others had no bands amplified). A: the *Agrobacterium*; WT: Wild-type; numbers 2, 3, 6, 7, 8, 9, 11 and 14: Positive plants from treatment 2; number 24: Positive plants from treatment 18; numbers 48 and 50: Positive plants from treatment 7, other numbers are untransformed plants.

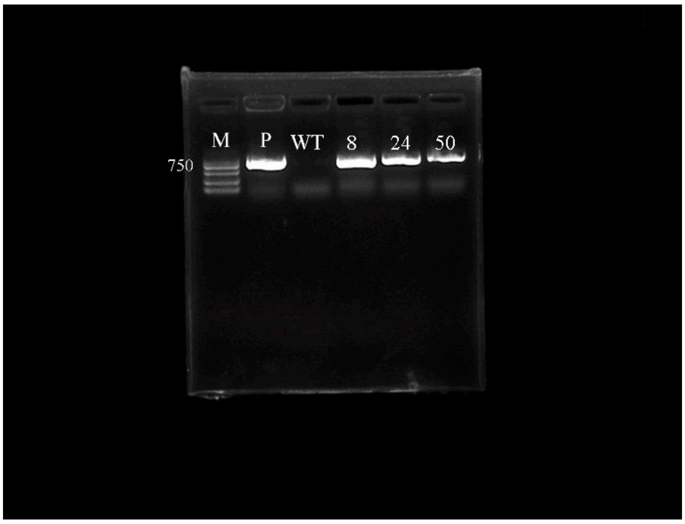

Figure S3 RT-PCR analysis of *GUS* expression in Wild-type plants and transgenic lines. M: Marker 2000, P: pCAMBIA1304, WT: Wild type, numbers 8, 24 and 50: Transgenic lines from treatment 2, treatment 18, and treatment 7, respectively;

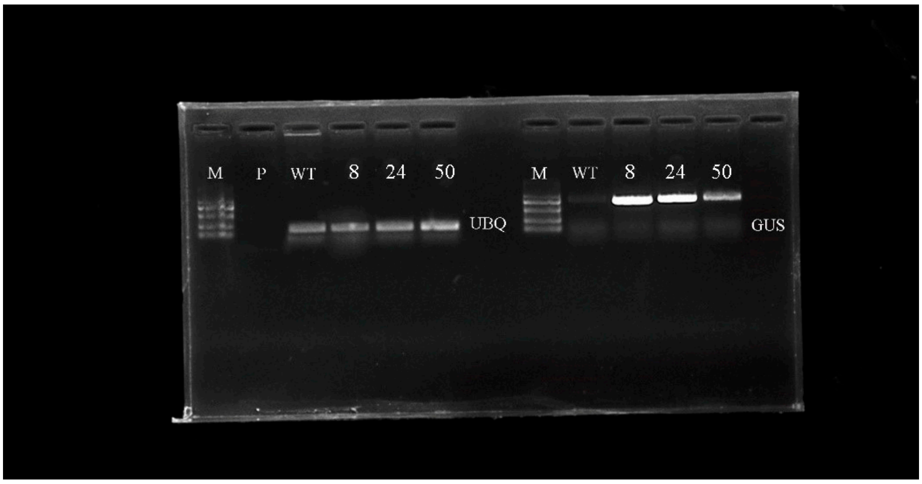

Figure S4 RT-PCR Result of *UBQ* and *GUS* in Wild-type plants and transgenic lines
M: Marker 2000, P: pCAMBIA1304, WT: Wild type, numbers 8, 24 and 50: Transgenic lines from treatment 2, treatment 18, and treatment 7, respectively;

Table S1. Effects of different hygromycin concentrations on petiole induction in callus

**Hygromycin concentration ($mg·L^{-1}$)**	**Bleaching rate (%)**	**Browning rate (%)**	**Callus induction rate (%)**	**Petiole growth state**
0.00	0.00	0.00	100.00±0.00a	Grow robustly
0.40	0.00	7.78±1.92d	92.22±1.92b	Growth inhibition
0.60	0.00	38.88±5.09c	61.11±5.09c	Petiole growth is slow
0.80	0.00	65.55±5.09b	34.44±5.09d	The petiole grows slowly and browns
1.00	100.00	0.00	0.00	The petiole stops growing, and the albino dies
2.00	0.00	100.00a	0.00	The petiole stops growing, browns and dies

The data are mean ± standard deviation, and different letters in the same column indicate significant differences between treatments ($P<0.05$), the same as below.
